# Distribution of Introns in Fungal Histone Genes

**DOI:** 10.1371/journal.pone.0016548

**Published:** 2011-01-27

**Authors:** Choong-Soo Yun, Hiromi Nishida

**Affiliations:** Agricultural Bioinformatics Research Unit, Graduate School of Agricultural and Life Sciences, University of Tokyo, Tokyo, Japan; University of California Riverside, United States of America

## Abstract

Saccharomycotina and Taphrinomycotina lack intron in their histone genes, except for an intron in one of histone H4 genes of *Yarrowia lipolytica*. On the other hand, Basidiomycota and Perizomycotina have introns in their histone genes. We compared the distributions of 81, 47, 79, and 98 introns in the fungal histone H2A, H2B, H3, and H4 genes, respectively. Based on the multiple alignments of the amino acid sequences of histones, we identified 19, 13, 31, and 22 intron insertion sites in the histone H2A, H2B, H3, and H4 genes, respectively. Surprisingly only one hot spot of introns in the histone H2A gene is shared between Basidiomycota and Perizomycotina, suggesting that most of introns of Basidiomycota and Perizomycotina were acquired independently. Our findings suggest that the common ancestor of Ascomycota and Basidiomycota maybe had a few introns in the histone genes. In the course of fungal evolution, Saccharomycotina and Taphrinomycotina lost the histone introns; Basidiomycota and Perizomycotina acquired other introns independently. In addition, most of the introns have sequence similarity among introns of phylogenetically close species, strongly suggesting that horizontal intron transfer events between phylogenetically distant species have not occurred recently in the fungal histone genes.

## Introduction

Eukaryotic genomic DNA is packaged with histone proteins to form chromatin [1]. The most fundamental repeating unit of chromatin is the nucleosome [2]. The nucleosome consists of the DNA wrapped around an octamer of histones (core histones) containing 2 copies each of histones H2A, H2B, H3, and H4. Generally eukaryotes have replication-dependent and independent histone genes [3]. Replication of the eukaryotic chromosomes requires the synthesis of histones to package the newly replicated DNA into chromatin. As cells progress from G1 to S phase in the cell cycle, the rate of histone gene transcription increases 3- to 5-fold, and the efficiency of histone pre-mRNA processing increases 8- to 10-fold, resulting in a 35-fold increase in histone protein levels [4], [5]. Based on the difference of transcription patterns during the cell cycle, we can identify whether a histone gene is replication-dependent or independent [6], [7]. Generally animal and plant replication-dependent histone genes are intronless. However, although animal replication-dependent histone genes lack a poly(A) signal, plant replication-dependent histone genes are polyadenylated [8].

**Figure 1:**
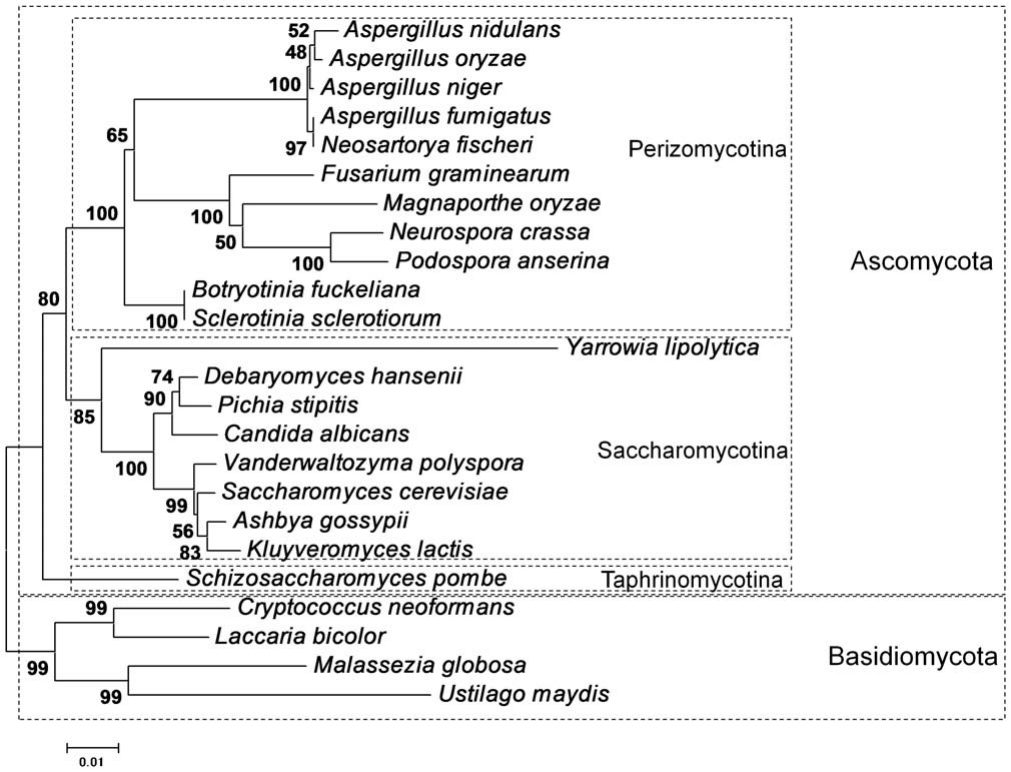
Phylogenetic relationships among the fungal species used in this analysis. Phylogenetic analysis was performed based on 1276 nucleotide sites of 18S rRNA gene without the gap/insertion sites. The neighbor-joining tree was reconstructed using the MEGA software [21]. The bootstrap was performed with 1000 replicates. The rate variation among sites was considered with gamma distributed rate (α = 1). The other default parameters were not changed.

Interestingly, histone genes of the filamentous ascomycetes *Aspergillus nidulans* and *Neurospora crassa* contain introns [9]–[11]. In addition, the replication-dependent histone genes of *Aspergillus nidulans* contain a poly(A) signal [9]. From the viewpoint of presence of poly(A) signal, the replication-dependent histone genes of those filamentous ascomycetes are more similar to those of plants than those of animals. However, from the viewpoint of presence of intron, the replication-dependent histone genes of those filamentous ascomycetes are different from those of both animals and plants. It is difficult to find similar intron-nucleotide sequences between introns from different organisms because nucleotide substitution rate of introns is higher than that of exons. Thus, main approach in the comparative studies of introns is to compare intron insertion sites among the different organisms. Stajich et al. analyzed intron insertion sites in 1161 sets of orthologous across eukaryotic species [12]. The result indicated that the fungal-animal ancestor was very intron rich and all modern fungi have experienced more intron loss than gain [12]. The purpose of this study is to compare the distributions of introns in fungal histone genes and discuss whether the ancestor of fungi has intron in the histone genes or not. The phylogenetic relationships among the fungal species used in this study is shown in Fig. 1.

## Results and Discussion

**Figure 2:**
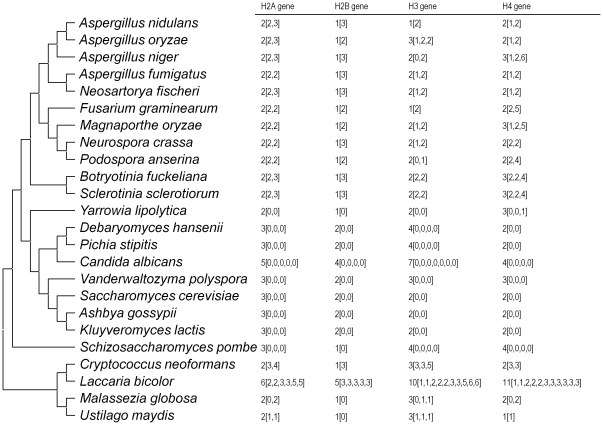
Number of core histone genes and introns in the gene. The numbers in parentheses indicate the number of introns within each gene. The phylogenetic topology is based on Fig. 1.

Phylogenetic tree based on histone H2B proteins shows that fungi are more closely related to animals than plants, which is consistent with the phylogeneny of organisms [13]. In each fungus used in this study, histone H2B gene has the lowest gene copy number among H2A, H2B, H3, and H4 histone genes (Fig. 2). On the other hand, in human and mouse, histone H2B gene has the highest gene copy number among replication-dependent H2A, H2B, H3, and H4 histone genes [14]. Thus, difference of the histone gene copy numbers maybe reflects on the phylogenetic lineage.

Ascomycetous yeasts (*Schizosaccharomyces* of Taphrinomycotina and Saccharomycotina) lack intron in their histone genes, except for an intron in one of the histone H4 genes of *Yarrowia lipolytica* (Fig. 2). On the other hand, Basidiomycota and Perizomycotina (filamentous ascomycetes) have introns in their histone genes (Fig. 2). After Ascomycota and Basidiomycota were branched off in the course of fungal evolution, Taphrinomycotina (“Archiascomycetes”) diverged prior to the separation of Saccharomycotina (“Hemiascomycetes”) and Perizomycotina (“Euascomycetes”) in the Ascomycota lineage [15]–[17]. Therefore, Saccharomycotina is phylogenetically more closely related to Perizomycotina than Taphrinomycotina. Why only Perizomycotina has introns in the histone genes of Ascomycota? We have no idea whether filamentous growth and yeast growth are related to presence and absence of introns in the histone genes of Ascomycota respectively or not. Because the basidomycetous yeast *Cryptococcus neoformans* has introns in its histone genes (Fig. 2). The bias of the intron distribution is not limited in the histone genes but also in the other genes [12].

If the common ancestor of fungal lineages had already had introns in its histone genes, the locations of most of Basidiomycota introns and Perizomycotina introns would be shared in the histone genes. In order to compare the intron insertion sites between Basidomycota and Perizomycotina, we identified 19, 13, 31, and 22 intron insertion sites in the histone H2A, H2B, H3, and H4 genes, respectively (Tables S1, S2, S3, and S4, Figs. S1, S2, S3, and S4). The histone H2A, H2B, H3, and H4 genes have 3, 3, 1, and 2 hot spots of introns of Perizomycotina, respectively (Tables S1, S2, S3, and S4). In addition, the histone H2A, H2B, H3, and H4 genes have 2, 2, 1, and 2 hot spots of Basidiomycota, respectively (Tables S1, S2, S3, and S4). Among those hot spots, only one site in histone H2A gene is shared between Basidiomycota and Perizomycotina. Except for the shared hot spot, Basidiomycota have 6 hot spots and Perizomycotina have 8 hot spots. Among the 6 hot spots of Basidiomycota, no site is shared with any introns of Perizomycotina. Among the 8 hot spots of Perizomycotina, only three sites (one in the H2A gene, one in the H2B gene, and one in the H3 gene; Tables S1, S2, and S3) are shared with some introns of Basidiomycota. These results strongly suggest that most of introns of Basidiomycota and Perizomycotina were acquired independently.

The intron insertion site in the histone H4_3 gene of *Yarrowia lipolytica* (belonging to Saccharomycotina) was shared with one of 2 hot spots in the histone H4 genes of Perizomycotina (Table S4). Considering that *Yarrowia lipolytica* branched off at the early stage of the Saccharomycotina evolution [18], it is suggested that the common ancestor of Perizomycotina and Saccharomycotina had an intron at the hot spot in histone H4 gene. In the course of evolution, most of Perizomycotina have maintained the intron; most of Saccharomycotina have lost it.

The distribution of the introns with nucleotide sequence similarity is so limited. We detected 134 intron-pairs with sequence similarity (Table S5). Among the 134 intron-pairs, 113 (84%) share the same insertion site in the same histone gene, 13 (9.7%) have different sites in the same histone gene, and 8 (6.0%) locate in different histone genes (Table S5). The similar sequence introns among the different species were found mainly between *Aspergillus fumigatus* and *Neosartorya fischeri* (28 of the 134, 21%), and between *Botryotinia fuckeliana* and *Sclerotinia sclerotiorum* (29 of the 134, 22%) (Table S5). Those results do not seem strange because *A. fumigatus* is phylogenetically more closely related to *N. fischeri* than to *A. nidulans*, *A. niger*, and *A. oryzae* [19] (Fig. 1) and *B. fuckeliana* is phylogenetically closely related to *S. sclerotiorum* [20] (Fig. 1). Therefore, it is strongly suggested that those similar sequence introns had already existed in the ancestor between *A. fumigatus* and *N. fischeri*, and in the ancestor between *B. fuckeliana* and *S. sclerotiorum*. Those introns have been inherited vertically.

The histone H2A_2 gene of *Aspergillus fumigatus* lacks intron at the position 17 (hot spot) (Table S1). However, the nucleotide sequence encoding 22 amino acids (from the 120th amino acid site to the 141st amino acid site, downstream from the intron insertion site) of histone H2A_2 of *A. fumigatus* has a sequence similarity with the 53-nt intron in the histone H2A_1 gene of *Neosartorya fischeri* (Fig. S1). The histone H4_1 gene of *A. fumigatus* and the histone H4_2 gene of *N. fischeri* have the three introns with sequence similarity at the same insertion site (Table S5). However, although the intron in the histone H4_2 gene of *A. fumigatus* has the same insertion site with that in the histone H4_1 gene of *N. fischeri*, the sequence lengths are so different (24-nt and 243-nt, Table S5). The 24-nt intron of *A. fumigatus* has a sequence similarity to the 3′-end region of the 243-nt intron of *N. fischeri*. The nucleotide sequence encoding 42 amino acids (from the N-terminal to the 42nd amino acid site, upstream from the intron insertion site) of histone H4_2 of *A. fumigatus* has a sequence similarity with the 243-nt intron of *N. fischeri* (Fig. S4). In order to elucidate those intron regions, additional biochemical studies are needed.

In addition, similar sequence introns were found within the *Laccaria bicolor* histone genes (62 of the 134, 46%) (Table S5). It suggests that histone gene containing introns had been duplicated frequently or homologus recombination occurred several times in the *Laccaria bicolor* genome.

In order to find similar sequences of the 335 introns in the fungal histone genes used in this study, we used the Fungi Genomes BLAST in the Fungal Genomes Central on the NCBI web site (http://www.ncbi.nlm.nih.gov/projects/genome/guide/fungi/) and performed the sequence-similarity search for the 102 fungal genome sequences. As the result, the distribution of most of the introns (except for some introns) is so limited among the phylogenetically closely related species (Table S6), strongly suggesting that horizontal intron transfer events between the phylogenetically distant species have not occurred recently in the fungal histone genes. As an exception, although *Aspergillus nidulans* H4_2 intron (88-nt) has a unique insertion site (Table S4), the sequence is found the most frequently among the different fungal genomes (Table S6).

## Methods

We compared the following 24 fungal genome sequences. Taphrinomycotina of Ascomycota (1 species); *Schizosaccharomyces pombe*. Saccharomycotina of Ascomycota (8 species); *Ashbya gossypii*, *Candida albicans*, *Debaryomyces hansenii*, *Kluyveromyces lactis*, *Pichia stipitis*, *Saccharomyces cerevisiae*, *Vanderwaltozyma polyspora*, and *Yarrowia lipolytica*. Perizomycotina of Ascomycota (11 species); *Aspergillus fumigatus*, *Aspergillus nidulans*, *Aspergillus niger*, *Aspergillus oryzae*, *Botryotinia fuckeliana*, *Fusarium graminearum*, *Magnaporthe oryzae*, *Neosartorya fischeri*, *Neurospora crassa*, *Podospora anserine*, and *Sclerotinia sclerotiorum*. Basidiomycota (4 species); *Cryptococcus neoformans*, *Laccaria bicolor*, *Malassezia globosa*, and *Ustilago maydis*. We extracted all core histones H2A, H2B, H3, and H4 genes from each fungal genome. The locations and the DNA lengths of introns were identified using the sequence viewer on the NCBI web site (http://www.ncbi.nlm.nih.gov/).

Amino acid sequences of the histones were multiple-aligned using the CLUSTAL W of MEGA software [21]. Based on the alignment, the insertion sites of the introns were compared. When an intron insertion site was shared among one third or more the introns within Basidiomycota and Perizomycotina, the insertion site is treated as hot spot of Basidiomycota and Perizomycotina, respectively.

We selected introns with nucleotide sequence similarity using the SSEARCH with Smith-Waterman similarity scores (http://fasta.bioch.virginia.edu/fasta_www2/fasta_list2.shtml) using 200 PRSS iterations [22] among the 335 introns used in this study. In this study, we selected the introns with *E*-value <0.02 as introns with nucleotide sequence similarity.

## Supporting Information

Figure S1 Alignment of fungal histone H2A proteins and the intron insertion site. The character “I” on the first row indicates the intron insertion site.
(TXT)

Figure S2 Alignment of fungal histone H2B proteins and the intron insertion site. The character “I” on the first row indicates the intron insertion site.
(TXT)

Figure S3 Alignment of fungal histone H3 proteins and the intron insertion site. The character “I” on the first row indicates the intron insertion site.
(TXT)

Figure S4 Alignment of fungal histone H4 proteins and the intron insertion site. The character “I” on the first row indicates the intron insertion site.
(TXT)

Table S1 Distribution of introns in fungal histone H2A genes.
(DOCX)

Table S2 Distribution of introns in fungal histone H2B genes.
(DOCX)

Table S3 Distribution of introns in fungal histone H3 genes.
(DOCX)

Table S4 Distribution of introns in fungal histone H4 genes.
(DOCX)

Table S5 Introns with sequence similarity. Yellow indicates the intron pair with different insertion sites in the same histone gene. Orange indicates the intron pair of different histone genes. The other indicates the intron pair with the same insertion site in the same histone gene.
(DOCX)

Table S6 Results (*E*-value <0.02) of the Fungi Genome BLAST in the Fungal Genomes Central on the NCBI web site for each intron used in this study. The hit sequence ID with the score (bits) and *E*-value are shown.
(TXT)
