# Supplementary material for: Distribution of Introns in Fungal Histone Genes
Source: PLoS One. 2011 Jan 27;6(1):e16548. doi: 10.1371/journal.pone.0016548 (PMC3029354; doi:10.1371/journal.pone.0016548)
Supplement: Table S1 — Distribution of introns in fungal histone H2A genes. (DOCX) [file pone.0016548.s005.docx]

| Table S1. Distribution of introns in fungal histone H2A genes | | | | | | | | | | | | | | | | | | | | |
| --- | --- | --- | --- | --- | --- | --- | --- | --- | --- | --- | --- | --- | --- | --- | --- | --- | --- | --- | --- | --- |
| Orgaism | Gene ID | Location of intron based on the alignment data (Fig. S1) and the length | | | | | | | | | | | | | | | | | | |
|  |  | 1 | 2 | 3 | 4 | 5* | 6^‡^ | 7 | 8 | 9 | 10^†^ | 11 | 12 | 13 | 14 | 15 | 16 | 17* | 18 | 19 |
| *Aspergillus nidulans* | *H2A_1* |  |  |  |  |  | 240 |  |  | 63 |  |  |  |  |  |  |  |  |  |  |
|  | *H2A_2* |  |  |  |  | 51 |  |  | 57 |  |  |  |  |  |  |  |  | 50 |  |  |
| *Aspergillus oryzae* | *H2A_1* |  |  |  |  |  | 312 |  |  | 80 |  |  |  |  |  |  |  |  |  |  |
|  | *H2A_2* |  |  |  |  | 55 |  |  | 51 |  |  |  |  |  |  |  |  | 54 |  |  |
| *Aspergillus niger* | *H2A_1* |  |  |  |  | 66 |  |  | 52 |  |  |  |  |  |  |  |  | 52 |  |  |
|  | *H2A_2* |  |  |  |  |  | 355 |  |  | 83 |  |  |  |  |  |  |  |  |  |  |
| *Aspergillus fumigatus* | *H2A_1* |  |  |  |  |  | 324 |  |  | 79 |  |  |  |  |  |  |  |  |  |  |
|  | *H2A_2* |  |  |  |  | 55 |  |  | 71 |  |  |  |  |  |  |  |  |  |  |  |
| *Neosartorya fischeri* | *H2A_1* |  |  |  |  | 56 |  |  | 69 |  |  |  |  |  |  |  |  | 53 |  |  |
|  | *H2A_2* |  |  |  |  |  | 322 |  |  | 81 |  |  |  |  |  |  |  |  |  |  |
| *Fusarium graminearum* | *H2A_1* |  |  |  |  |  | 379 |  |  |  |  |  |  | 56 |  |  |  |  |  |  |
|  | *H2A_2* |  |  |  |  | 79 |  |  |  |  |  |  |  |  |  |  |  | 51 |  |  |
| *Magnaporthe oryzae* | *H2A_1* |  |  |  |  | 161 |  |  |  |  |  |  |  |  |  |  |  | 74 |  |  |
|  | *H2A_2* |  |  |  |  |  | 514 |  |  |  |  |  |  | 97 |  |  |  |  |  |  |
| *Neurospora crassa* | *H2A_1* |  |  |  |  | 122 |  |  |  |  |  |  |  |  |  |  |  | 64 |  |  |
|  | *H2A_2* |  |  |  |  |  | 491 |  |  |  |  |  |  | 109 |  |  |  |  |  |  |
| *Podospora anserine* | *H2A_1* |  |  |  |  |  | 368 |  |  |  |  |  |  | 77 |  |  |  |  |  |  |
|  | *H2A_2* |  |  |  |  | 149 |  |  |  |  |  |  |  |  |  |  |  | 55 |  |  |
| *Botryotinia fuckeliana* | *H2A_1* |  |  |  |  | 126 |  |  | 57 |  |  |  |  |  |  |  |  | 51 |  |  |
|  | *H2A_2* |  |  |  |  |  | 402 |  |  |  |  |  |  | 56 |  |  |  |  |  |  |
| *Sclerotinia sclerotiorum* | *H2A_1* |  |  |  |  | 130 |  |  | 79 |  |  |  |  |  |  |  |  | 53 |  |  |
|  | *H2A_2* |  |  |  |  |  | 399 |  |  |  |  |  |  | 56 |  |  |  |  |  |  |
| *Cryptococcus neoformans* | *H2A_1* |  |  |  |  |  | 66 | 90 |  |  |  |  | 50 |  |  |  |  | 52 |  |  |
|  | *H2A_2* |  |  |  |  |  | 54 |  |  |  |  | 59 |  |  |  |  |  |  | 51 |  |
| *Laccaria bicolor* | *H2A_1* | 47 |  | 61 |  |  |  |  |  | 44 |  |  |  |  | 53 |  | 49 |  |  |  |
|  | *H2A_2* |  |  |  |  |  | 55 |  |  |  |  |  | 52 |  |  |  |  |  |  |  |
|  | *H2A_3* |  |  |  | 56 |  |  |  |  |  | 53 |  |  |  |  |  |  |  |  |  |
|  | *H2A_4* |  |  |  |  |  |  |  |  |  | 54 |  |  |  | 52 |  | 51 |  |  |  |
|  | *H2A_5* |  |  |  |  |  |  |  |  |  | 53 |  |  |  |  | 51 |  | 52 |  |  |
|  | *H2A_6* |  | 118 |  |  |  |  |  |  |  | 56 |  |  |  | 50 |  |  | 49 |  | 57 |
| *Malassezia globosa* | *H2A_1* |  |  |  |  |  | 45 |  |  |  |  |  | 48 |  |  |  |  |  |  |  |
|  | *H2A_2* |  |  |  |  |  |  |  |  |  |  |  |  |  |  |  |  |  |  |  |
| *Ustilago maydis* | *H2A_1* |  |  |  |  |  | 569 |  |  |  |  |  |  |  |  |  |  |  |  |  |
|  | *H2A_2* |  |  |  |  |  |  |  |  |  | 94 |  |  |  |  |  |  |  |  |  |
| Number of introns |  | 1 | 1 | 1 | 1 | 11 | 16 | 1 | 7 | 6 | 5 | 1 | 3 | 6 | 3 | 1 | 2 | 13 | 1 | 1 |
|  |  |  |  |  |  |  |  |  |  |  |  |  |  |  |  |  |  |  |  |  |
| *hot spot of Perizomycotina, †hot spot of Basidiomycota, ‡hot spot of both Perizomycotina and Basidiomycota. | | | | | | | | | | | | | | | | | | | | |
