# Supplementary material for: Distribution of Introns in Fungal Histone Genes
Source: PLoS One. 2011 Jan 27;6(1):e16548. doi: 10.1371/journal.pone.0016548 (PMC3029354; doi:10.1371/journal.pone.0016548)
Supplement: Table S2 — Distribution of introns in fungal histone H2B genes. (DOCX) [file pone.0016548.s006.docx]

| Table S2. Distribution of introns in fungal histone H2B genes | | | | | | | | | | | | | | |
| --- | --- | --- | --- | --- | --- | --- | --- | --- | --- | --- | --- | --- | --- | --- |
| Orgaism | Gene ID | Location of intron based on the alignment data (Fig. S2) and the length | | | | | | | | | | | | |
|  |  | 1 | 2 | 3^†^ | 4 | 5 | 6* | 7^†^ | 8 | 9* | 10 | 11* | 12 | 13 |
| *Aspergillus nidulans* | *H2B_1* |  |  |  |  |  | 52 |  |  | 52 |  | 46 |  |  |
| *Aspergillus oryzae* | *H2B_1* |  |  |  |  |  |  |  |  | 51 |  | 48 |  |  |
| *Aspergillus niger* | *H2B_1* |  |  |  |  |  | 54 |  |  | 55 |  | 50 |  |  |
| *Aspergillus fumigatus* | *H2B_1* |  |  |  |  |  | 50 |  |  | 53 |  | 50^c^ |  |  |
| *Neosartorya fischeri* | *H2B_1* |  |  |  |  |  | 50 |  |  | 52 |  | 51^c^ |  |  |
| *Fusarium graminearum* | *H2B_1* |  |  |  |  |  | 76 |  |  | 51 |  |  |  |  |
| *Magnaporthe oryzae* | *H2B_1* |  |  |  |  |  | 102 |  |  | 81 |  |  |  |  |
| *Neurospora crassa* | *H2B_1* |  |  |  |  |  | 141 |  |  | 55 |  | 68 |  |  |
| *Podospora anserine* | *H2B_1* |  |  |  |  |  | 87 |  |  | 67 |  |  |  |  |
| *Botryotinia fuckeliana* | *H2B_1* | 58 |  |  |  |  | 83 |  |  |  |  | 50 |  |  |
| *Sclerotinia sclerotiorum* | *H2B_1* | 55 |  |  |  |  | 95 |  |  |  |  | 53 |  |  |
| *Cryptococcus neoformans* | *H2B_1* |  | 57 |  |  |  |  |  | 55 |  |  |  | 50 |  |
| *Laccaria bicolor* | *H2B_1* |  |  | 13 |  | 767 |  | 49 |  |  |  |  |  |  |
|  | *H2B_2* |  |  |  |  |  | 49 |  |  |  | 58 |  |  | 53 |
|  | *H2B_3* |  |  |  |  |  | 65 |  |  |  | 56 |  |  | 53 |
|  | *H2B_4* |  |  | 13 |  | 767 |  | 49 |  |  |  |  |  |  |
|  | *H2B_5* |  |  | 13 | 767 |  |  | 49 |  |  |  |  |  |  |
| *Malassezia globosa* | *H2B_1* |  |  |  |  |  |  |  |  |  |  |  |  |  |
| *Ustilago maydis* | *H2B_1* |  |  |  |  |  |  |  |  |  |  |  |  |  |
| Number of introns |  | 2 | 1 | 3 | 1 | 2 | 12 | 3 | 1 | 9 | 2 | 8 | 1 | 2 |
|  |  |  |  |  |  |  |  |  |  |  |  |  |  |  |
| *hot spot of Perizomycotina, †hot spot of Basidiomycota. | | | | | | | | | | | | | | |
