# Supplementary material for: Distribution of Introns in Fungal Histone Genes
Source: PLoS One. 2011 Jan 27;6(1):e16548. doi: 10.1371/journal.pone.0016548 (PMC3029354; doi:10.1371/journal.pone.0016548)
Supplement: Table S3 — Distribution of introns in fungal histone H3 genes. (DOCX) [file pone.0016548.s007.docx]

| Table S3. Distribution of introns in fungal histone H3 genes | | | | | | | | | | | | | | | | | | | | | | | | | | | | | | | | |
| --- | --- | --- | --- | --- | --- | --- | --- | --- | --- | --- | --- | --- | --- | --- | --- | --- | --- | --- | --- | --- | --- | --- | --- | --- | --- | --- | --- | --- | --- | --- | --- | --- |
| Orgaism | Gene ID | Location of intron based on the alignment data (Fig. S3) and the length | | | | | | | | | | | | | | | | | | | | | | | | | | | | | | |
|  |  | 1 | 2 | 3 | 4 | 5 | 6 | 7 | 8 | 9 | 10 | 11 | 12* | 13 | 14 | 15 | 16 | 17 | 18 | 19 | 20 | 21 | 22 | 23^†^ | 24 | 25 | 26 | 27 | 28 | 29 | 30 | 31 |
| *Aspergillus nidulans* | *H3_1* |  |  |  | 79 |  |  |  |  |  |  |  |  |  |  |  |  |  | 52 |  |  |  |  |  |  |  |  |  |  |  |  |  |
| *Aspergillus oryzae* | *H3_1* |  |  | 113 |  |  |  |  |  |  |  |  |  |  |  |  |  |  | 59 |  |  |  |  |  |  |  |  |  |  |  |  |  |
|  | *H3_2* |  |  |  |  |  |  |  | 63 |  |  |  |  |  |  |  |  |  |  |  |  |  |  |  |  |  |  |  |  |  |  | 35 |
|  | *H3_3* |  |  |  |  |  |  |  |  |  |  |  | 77 |  |  |  |  |  |  |  |  |  |  |  |  |  |  |  |  |  |  |  |
| *Aspergillus niger* | *H3_1* |  |  | 109 |  |  |  |  |  |  |  |  |  |  |  |  |  |  | 69 |  |  |  |  |  |  |  |  |  |  |  |  |  |
|  | *H3_2* |  |  |  |  |  |  |  |  |  |  |  |  |  |  |  |  |  |  |  |  |  |  |  |  |  |  |  |  |  |  |  |
| *Aspergillus fumigatus* | *H3_1* |  |  | 129 |  |  |  |  |  |  |  |  |  |  |  |  |  |  | 53 |  |  |  |  |  |  |  |  |  |  |  |  |  |
|  | *H3_2* |  |  |  |  |  |  |  |  |  |  |  | 74 |  |  |  |  |  |  |  |  |  |  |  |  |  |  |  |  |  |  |  |
| *Neosartorya fischeri* | *H3_1* |  |  | 126 |  |  |  |  |  |  |  |  |  |  |  |  |  |  | 56 |  |  |  |  |  |  |  |  |  |  |  |  |  |
|  | *H3_2* |  |  |  |  |  |  |  |  |  |  |  | 75 |  |  |  |  |  |  |  |  |  |  |  |  |  |  |  |  |  |  |  |
| *Fusarium graminearum* | *H3_1* |  |  |  |  |  |  |  |  |  | 53 |  |  |  |  |  |  |  |  |  |  |  | 58 |  |  |  |  |  |  |  |  |  |
| *Magnaporthe oryzae* | *H3_1* |  |  |  |  |  |  |  |  |  |  |  |  |  |  |  |  |  |  |  |  |  | 87 |  |  |  |  |  |  |  | 61 |  |
|  | *H3_2* |  |  |  |  |  |  |  |  |  |  |  | 88 |  |  |  |  |  |  |  |  |  |  |  |  |  |  |  |  |  |  |  |
| *Neurospora crassa* | *H3_1* |  |  |  |  |  |  |  |  |  |  |  |  |  |  |  |  |  |  |  |  |  | 67 |  |  |  |  |  |  |  |  |  |
|  | *H3_2* |  |  |  |  |  |  |  |  |  |  |  | 145 |  |  |  |  |  |  |  |  |  | 83 |  |  |  |  |  |  |  |  |  |
| *Podospora anserine* | *H3_1* |  |  |  |  |  |  |  |  |  |  |  |  |  |  |  |  | 81 |  |  |  |  |  |  |  |  |  |  |  |  |  |  |
|  | *H3_2* |  |  |  |  |  |  |  |  |  |  |  |  |  |  |  |  |  |  |  |  |  |  |  |  |  |  |  |  |  |  |  |
| *Botryotinia fuckeliana* | *H3_1* |  |  | 164 |  |  |  |  |  |  |  |  |  |  |  |  |  | 51 |  |  |  |  |  |  |  |  |  |  |  |  |  |  |
|  | *H3_2* |  |  |  |  |  |  |  |  |  |  |  | 144 |  |  |  |  |  |  |  |  |  | 59 |  |  |  |  |  |  |  |  |  |
| *Sclerotinia sclerotiorum* | *H3_1* |  |  | 158 |  |  |  |  |  |  |  |  |  |  |  |  |  | 49 |  |  |  |  |  |  |  |  |  |  |  |  |  |  |
|  | *H3_2* |  |  |  |  |  |  |  |  |  |  |  | 147 |  |  |  |  |  |  |  |  |  | 59 |  |  |  |  |  |  |  |  |  |
| *Cryptococcus neoformans* | *H3_1* |  |  |  |  |  |  |  |  |  |  |  |  | 162 |  |  |  |  |  |  |  |  | 170 |  |  |  | 155 |  |  |  |  |  |
|  | *H3_2* | 79 | 303 |  |  |  |  | 49 |  |  |  |  |  |  |  |  |  |  |  | 52 |  |  |  |  |  |  | 53 |  |  |  |  |  |
|  | *H3_3* |  |  |  |  |  |  | 62 |  |  |  |  |  |  |  |  |  |  |  | 55 |  |  |  | 61 |  |  |  |  |  |  |  |  |
| *Laccaria bicolor* | *H3_1* |  |  |  |  | 57 |  |  |  |  |  |  |  |  | 51 |  |  |  |  |  |  |  |  |  | 61 |  |  |  |  |  |  |  |
|  | *H3_2* |  |  |  |  |  |  |  |  |  |  |  | 137 |  |  |  |  |  |  |  | 482 | 349 |  | 111 |  |  |  | 716 |  |  |  |  |
|  | *H3_3* |  |  |  |  |  | 175 |  |  | 17 |  |  |  |  |  |  |  |  |  |  |  |  |  | 70^d^ |  |  |  |  |  |  |  |  |
|  | *H3_4* |  |  |  |  |  |  |  |  |  |  |  |  |  |  |  |  |  |  |  |  |  |  |  | 68^d^ |  |  |  |  |  |  |  |
|  | *H3_5* |  | 85 |  |  | 58 |  |  |  |  |  |  |  |  | 111 |  |  |  |  |  |  |  |  | 130 |  |  |  |  | 91 | 59 |  |  |
|  | *H3_6* |  |  |  |  |  |  |  |  |  |  |  |  | 51 |  |  |  |  |  |  |  |  |  | 50 |  |  |  |  |  |  |  |  |
|  | *H3_7* |  |  |  |  |  |  |  |  |  |  |  |  |  |  | 30 |  |  |  |  |  |  |  | 62 |  |  |  |  |  |  |  |  |
|  | *H3_8* |  |  |  |  |  |  |  |  |  |  |  |  |  |  |  |  |  |  |  |  |  |  | 58 |  |  |  |  |  |  |  |  |
|  | *H3_9* |  | 69 |  |  | 58 |  |  |  |  |  |  |  |  | 101 |  |  |  |  |  |  |  |  | 58 |  |  |  |  | 111 | 50 |  |  |
|  | *H3_10* |  |  |  |  |  |  |  |  |  |  |  |  |  |  |  |  |  |  |  |  |  | 52 |  |  |  | 51 |  |  |  |  |  |
| *Malassezia globosa* | *H3_1* |  |  |  |  |  |  |  |  |  |  |  |  |  |  |  |  |  |  |  |  |  |  |  |  |  |  |  |  |  |  |  |
|  | *H3_2* |  |  |  |  |  |  |  |  |  |  |  |  |  |  |  | 48 |  |  |  |  |  |  |  |  |  |  |  |  |  |  |  |
|  | *H3_3* |  |  |  |  |  |  |  |  |  |  | 40 |  |  |  |  |  |  |  |  |  |  |  |  |  |  |  |  |  |  |  |  |
| *Ustilago maydis* | *H3_1* |  |  |  |  |  | 117 |  |  |  |  |  |  |  |  |  |  |  |  |  |  |  |  |  |  |  |  |  |  |  |  |  |
|  | *H3_2* |  |  |  |  |  |  |  |  |  |  |  |  |  |  |  |  |  |  |  |  |  |  |  |  | 109 |  |  |  |  |  |  |
|  | *H3_3* |  |  |  |  |  |  |  |  |  |  |  |  |  |  |  |  |  |  |  |  |  |  |  |  |  | 144 |  |  |  |  |  |
| Number of introns |  | 1 | 3 | 6 | 1 | 3 | 2 | 2 | 1 | 1 | 1 | 1 | 8 | 2 | 3 | 1 | 1 | 3 | 5 | 2 | 1 | 1 | 8 | 8 | 2 | 1 | 4 | 1 | 2 | 2 | 1 | 1 |
|  |  |  |  |  |  |  |  |  |  |  |  |  |  |  |  |  |  |  |  |  |  |  |  |  |  |  |  |  |  |  |  |  |
| *hot spot of Perizomycotina, †hot spot of Basidiomycota. | | | | | | | | | | | | | | | | | | | | | | | | | | | | | | | | |
