# Supplementary material for: Distribution of Introns in Fungal Histone Genes
Source: PLoS One. 2011 Jan 27;6(1):e16548. doi: 10.1371/journal.pone.0016548 (PMC3029354; doi:10.1371/journal.pone.0016548)
Supplement: Table S4 — Distribution of introns in fungal histone H4 genes. (DOCX) [file pone.0016548.s008.docx]

| Table S4. Distribution of introns in fungal histone H4 genes | | | | | | | | | | | | | | | | | | | | | | | |
| --- | --- | --- | --- | --- | --- | --- | --- | --- | --- | --- | --- | --- | --- | --- | --- | --- | --- | --- | --- | --- | --- | --- | --- |
| Orgaism | Gene ID | Location of intron based on the alignment data (Fig. S4) and the length | | | | | | | | | | | | | | | | | | | | | |
|  |  | 1* | 2 | 3 | 4^†^ | 5 | 6 | 7 | 8^†^ | 9 | 10 | 11 | 12* | 13 | 14 | 15 | 16 | 17 | 18 | 19 | 20 | 21 | 22 |
| *Aspergillus nidulans* | *H4_1* | 166 |  |  |  |  |  |  |  |  |  |  |  |  |  |  |  |  |  |  |  |  |  |
|  | *H4_2* |  | 88 |  |  |  |  |  |  |  |  |  |  |  |  |  |  |  |  |  |  | 55 |  |
| *Aspergillus oryzae* | *H4_1* | 72 |  |  |  |  |  |  |  |  |  |  |  |  |  |  |  |  |  |  |  | 59 |  |
|  | *H4_2* | 222 |  |  |  |  |  |  |  |  |  |  |  |  |  |  |  |  |  |  |  |  |  |
| *Aspergillus niger* | *H4_1* | 245 |  |  |  |  |  |  |  |  |  |  |  |  |  |  |  |  |  |  |  |  |  |
|  | *H4_2* | 103 |  |  |  |  |  |  |  |  |  |  |  |  |  |  |  |  |  |  |  | 59 |  |
|  | *H4_3* |  |  |  |  | 85 |  |  |  | 58 | 87 |  |  | 51 |  |  |  |  | 67 |  |  | 29 |  |
| *Aspergillus fumigatus* | *H4_1* | 71 |  |  |  |  |  |  |  |  |  |  |  |  |  |  |  |  |  |  |  | 55 |  |
|  | *H4_2* | 24 |  |  |  |  |  |  |  |  |  |  |  |  |  |  |  |  |  |  |  |  |  |
| *Neosartorya fischeri* | *H4_1* | 243 |  |  |  |  |  |  |  |  |  |  |  |  |  |  |  |  |  |  |  |  |  |
|  | *H4_2* | 73 |  |  |  |  |  |  |  |  |  |  |  |  |  |  |  |  |  |  |  | 53 |  |
| *Fusarium graminearum* | *H4_1* |  |  |  |  | 61 |  |  |  | 37 | 55 |  |  | 59 |  |  |  |  |  | 55 |  |  |  |
|  | *H4_2* | 63 |  |  |  |  |  |  |  |  |  |  | 58 |  |  |  |  |  |  |  |  |  |  |
| *Magnaporthe oryzae* | *H4_1* |  |  |  |  |  |  |  |  |  |  |  | 81 |  |  |  |  |  |  |  |  |  |  |
|  | *H4_2* |  |  |  |  | 97 |  |  |  | 96 | 63 |  |  | 69 |  |  |  |  |  | 97 |  |  |  |
|  | *H4_3* | 214 |  |  |  |  |  |  |  |  |  |  | 64 |  |  |  |  |  |  |  |  |  |  |
| *Neurospora crassa* | *H4_1* | 69 |  |  |  |  |  |  |  |  |  |  | 68 |  |  |  |  |  |  |  |  |  |  |
|  | *H4_2* | 316 |  |  |  |  |  |  |  |  |  |  | 65 |  |  |  |  |  |  |  |  |  |  |
| *Podospora anserine* | *H4_1* | 297 |  |  |  |  |  |  |  |  |  |  | 60 |  |  |  |  |  |  |  |  |  |  |
|  | *H4_2* |  |  |  |  |  |  |  |  | 69 | 262 |  |  | 66 |  |  |  |  |  | 55 |  |  |  |
| *Botryotinia fuckeliana* | *H4_1* | 98 |  |  |  |  |  |  |  |  |  |  | 55 |  |  |  |  |  |  |  |  |  |  |
|  | *H4_2* |  |  |  |  | 136 |  |  |  |  | 51 |  |  | 49 |  |  |  |  |  | 58 |  |  |  |
|  | *H4_3* | 261 |  |  |  |  |  |  |  |  |  |  | 97 |  |  |  |  |  |  |  |  |  |  |
| *Sclerotinia sclerotiorum* | *H4_1* | 237 |  |  |  |  |  |  |  |  |  |  | 96 |  |  |  |  |  |  |  |  |  |  |
|  | *H4_2* | 108 |  |  |  |  |  |  |  |  |  |  | 52 |  |  |  |  |  |  |  |  |  |  |
|  | *H4_3* |  |  |  |  | 139 |  |  |  |  | 51 |  |  | 49 |  |  |  |  |  | 64 |  |  |  |
| *Yarrowia lipolytica* | *H4_3* | 79 |  |  |  |  |  |  |  |  |  |  |  |  |  |  |  |  |  |  |  |  |  |
| *Cryptococcus neoformans* | *H4_1* |  |  |  |  |  |  | 54 |  |  |  | 52 |  |  |  |  | 48 |  |  |  |  |  |  |
|  | *H4_2* |  |  |  |  |  |  | 55 |  |  |  | 51 |  |  |  |  | 53 |  |  |  |  |  |  |
| *Laccaria bicolor* | *H4_1* |  |  |  | 54 |  |  |  | 52 |  |  |  |  |  |  | 56 |  |  |  |  |  |  |  |
|  | *H4_2* |  |  |  |  |  |  |  |  |  |  |  |  | 55 |  |  |  |  |  |  |  |  |  |
|  | *H4_3* |  |  |  |  |  |  |  |  |  |  |  |  |  | 45 |  |  | 427 |  |  |  |  |  |
|  | *H4_4* |  |  |  | 54 |  |  |  | 51 |  |  |  |  | 55 |  |  |  |  |  |  |  |  |  |
|  | *H4_5* |  |  |  | 55 |  |  |  | 51 |  |  |  |  | 57 |  |  |  |  |  |  |  |  |  |
|  | *H4_6* |  |  |  | 55 |  |  |  | 61 |  |  |  |  |  |  |  |  |  |  |  |  |  |  |
|  | *H4_7* |  |  |  | 55 |  |  |  | 61 |  |  |  |  |  |  | 53 |  |  |  |  |  |  |  |
|  | *H4_8* |  |  |  |  |  |  |  |  |  |  |  |  |  | 45 |  |  |  |  |  | 443 |  |  |
|  | *H4_9* |  |  |  |  |  |  |  | 51 |  |  |  |  |  |  |  |  |  |  |  |  |  |  |
|  | *H4_10* |  |  |  | 54 |  |  |  | 53 |  |  |  |  |  |  | 51 |  |  |  |  |  |  |  |
|  | *H4_11* |  |  |  | 55 |  |  |  | 60 |  |  |  |  |  |  | 53 |  |  |  |  |  |  |  |
| *Malassezia globosa* | *H4_1* |  |  |  |  |  |  |  |  |  |  |  |  |  |  |  |  |  |  |  |  |  |  |
|  | *H4_2* |  |  | 41 |  |  |  |  |  |  |  |  |  |  |  |  |  |  |  |  |  |  | 27 |
| *Ustilago maydis* | *H4_1* |  |  |  |  |  | 102 |  |  |  |  |  |  |  |  |  |  |  |  |  |  |  |  |
| Number of introns |  | 19 | 1 | 1 | 7 | 5 | 1 | 2 | 8 | 4 | 6 | 2 | 10 | 9 | 2 | 4 | 2 | 1 | 1 | 5 | 1 | 6 | 1 |
|  |  |  |  |  |  |  |  |  |  |  |  |  |  |  |  |  |  |  |  |  |  |  |  |
| *hot spot of Perizomycotina, †hot spot of Basidiomycota. | | | | | | | | | | | | | | | | | | | | | | | |
