# Supplementary material for: Distribution of Introns in Fungal Histone Genes
Source: PLoS One. 2011 Jan 27;6(1):e16548. doi: 10.1371/journal.pone.0016548 (PMC3029354; doi:10.1371/journal.pone.0016548)
Supplement: Table S5 — Introns with sequence similarity. Yellow indicates the intron pair with different insertion sites in the same histone gene. Orange indicates the intron pair of different histone genes. The other indicates the intron pair with the same insertion site in the same histone gene. (DOCX) [file pone.0016548.s009.docx]

| Table S5. Introns with sequence similarity |  |  |  |
| --- | --- | --- | --- |
| Quary intron (length) | Hit intron (length) | Smith-Waterman score | *E*-value |
| Aspergillus_fumigatus_H2A_1_Intron_1/2_(324) | Neosartorya_fischeri_H2A_2_Intron_1/2_(322) | 1276 | 1.40E-89 |
| Aspergillus_fumigatus_H2A_1_Intron_2/2_(79) | Neosartorya_fischeri_H2A_2_Intron_2/2_(81) | 296 | 3.60E-01 |
| Aspergillus_fumigatus_H2A_2_Intron_1/2_(55) | Neosartorya_fischeri_H2A_1_Intron_1/3_(56) | 151 | 1.20E-06 |
| Aspergillus_fumigatus_H2A_2_Intron_2/2_(71) | Neosartorya_fischeri_H2A_1_Intron_2/3_(69) | 244 | 1.40E-13 |
| Botryotinia_fuckeliana_H2A_1_Intron_2/3_(57) | Sclerotinia_sclerotiorum_H2A_1_Intron_2/3_(79) | 93 | 0.016 |
| Botryotinia_fuckeliana_H2A_2_Intron_1/2_(402) | Sclerotinia_sclerotiorum_H2A_2_Intron_1/2_(399) | 898 | 6.00E-58 |
| Botryotinia_fuckeliana_H2A_2_Intron_2/2_(56) | Sclerotinia_sclerotiorum_H2A_2_Intron_2/2_(56) | 91 | 0.018 |
| Neosartorya_fischeri_H2A_1_Intron_1/3_(56) | Aspergillus_fumigatus_H2A_2_Intron_1/2_(55) | 151 | 1.00E-07 |
| Neosartorya_fischeri_H2A_1_Intron_2/3_(69) | Aspergillus_fumigatus_H2A_2_Intron_2/2_(71) | 244 | 1.40E-12 |
| Neosartorya_fischeri_H2A_2_Intron_1/2_(322) | Aspergillus_fumigatus_H2A_1_Intron_1/2_(324) | 1276 | 7.80E-102 |
| Neosartorya_fischeri_H2A_2_Intron_2/2_(81) | Aspergillus_fumigatus_H2A_1_Intron_2/2_(79) | 296 | 2.50E-17 |
| Neurospora_crassa_H2A_1_Intron_1/2_(122) | Laccaria_bicolor_H2B_5_Intron_2/3_(767) | 100 | 0.011 |
| Neurospora_crassa_H2A_1_Intron_1/2_(122) | Laccaria_bicolor_H2B_1_Intron_2/3_(767) | 99 | 0.013 |
| Neurospora_crassa_H2A_1_Intron_1/2_(122) | Laccaria_bicolor_H2B_4_Intron_2/3_(767) | 99 | 0.013 |
| Sclerotinia_sclerotiorum_H2A_2_Intron_1/2_(399) | Botryotinia_fuckeliana_H2A_2_Intron_1/2_(402) | 898 | 9.20E-60 |
| Sclerotinia_sclerotiorum_H2A_2_Intron_2/2_(56) | Botryotinia_fuckeliana_H2A_2_Intron_2/2_(56) | 91 | 0.0073 |
| Laccaria_bicolor_H2A_3_Intron_2/2_(53) | Laccaria_bicolor_H2A_4_Intron_1/3_(54) | 159 | 3.70E-06 |
| Laccaria_bicolor_H2A_4_Intron_1/3_(54) | Laccaria_bicolor_H2A_3_Intron_2/2_(53) | 159 | 4.40E-09 |
| Ustilago_maydis_H2A_1_Intron_1/1_(569) | Botryotinia_fuckeliana_H3_1_Intron_1/2_(164) | 105 | 0.016 |
| Aspergillus_fumigatus_H2B_1_Intron_1/3_(50) | Neosartorya_fischeri_H2B_1_Intron_1/3_(50) | 169 | 9.10E-10 |
| Aspergillus_fumigatus_H2B_1_Intron_2/3_(53) | Neosartorya_fischeri_H2B_1_Intron_2/3_(52) | 199 | 5.50E-10 |
| Aspergillus_fumigatus_H2B_1_Intron_3/3_(50) | Neosartorya_fischeri_H2B_1_Intron_3/3_(51) | 200 | 7.90E-12 |
| Botryotinia_fuckeliana_H2B_1_Intron_1/3_(58) | Sclerotinia_sclerotiorum_H2B_1_Intron_1/3_(55) | 92 | 0.009 |
| Botryotinia_fuckeliana_H2B_1_Intron_2/3_(83) | Sclerotinia_sclerotiorum_H2B_1_Intron_2/3_(95) | 206 | 1.20E-10 |
| Botryotinia_fuckeliana_H2B_1_Intron_3/3_(50) | Sclerotinia_sclerotiorum_H2B_1_Intron_3/3_(53) | 99 | 0.0037 |
| Neosartorya_fischeri_H2B_1_Intron_1/3_(50) | Aspergillus_fumigatus_H2B_1_Intron_1/3_(50) | 169 | 3.00E-09 |
| Neosartorya_fischeri_H2B_1_Intron_2/3_(52) | Aspergillus_fumigatus_H2B_1_Intron_2/3_(53) | 199 | 2.50E-10 |
| Neosartorya_fischeri_H2B_1_Intron_3/3_(51) | Aspergillus_fumigatus_H2B_1_Intron_3/3_(50) | 200 | 5.30E-12 |
| Neosartorya_fischeri_H2B_1_Intron_3/3_(51) | Laccaria_bicolor_H2A_1_Intron_4/5_(53) | 91 | 0.0078 |
| Sclerotinia_sclerotiorum_H2B_1_Intron_2/3_(95) | Botryotinia_fuckeliana_H2B_1_Intron_2/3_(83) | 206 | 2.60E-09 |
| Laccaria_bicolor_H2B_1_Intron_1/3_(13) | Laccaria_bicolor_H2B_4_Intron_1/3_(13) | 65 | 0.011 |
| Laccaria_bicolor_H2B_1_Intron_1/3_(13) | Laccaria_bicolor_H2B_5_Intron_1/3_(13) | 65 | 0.011 |
| Laccaria_bicolor_H2B_1_Intron_2/3_(767) | Laccaria_bicolor_H2B_4_Intron_2/3_(767) | 3799 | 0 |
| Laccaria_bicolor_H2B_1_Intron_2/3_(767) | Laccaria_bicolor_H2B_5_Intron_2/3_(767) | 3639 | 0 |
| Laccaria_bicolor_H2B_1_Intron_3/3_(49) | Laccaria_bicolor_H2B_4_Intron_3/3_(49) | 245 | 7.30E-17 |
| Laccaria_bicolor_H2B_1_Intron_3/3_(49) | Laccaria_bicolor_H2B_5_Intron_3/3_(49) | 245 | 7.30E-17 |
| Laccaria_bicolor_H2B_2_Intron_3/3_(53) | Laccaria_bicolor_H2B_4_Intron_2/3_(767) | 99 | 0.0054 |
| Laccaria_bicolor_H2B_2_Intron_3/3_(53) | Laccaria_bicolor_H2B_1_Intron_2/3_(767) | 99 | 0.0054 |
| Laccaria_bicolor_H2B_4_Intron_1/3_(13) | Laccaria_bicolor_H2B_1_Intron_1/3_(13) | 65 | 0.011 |
| Laccaria_bicolor_H2B_4_Intron_1/3_(13) | Laccaria_bicolor_H2B_5_Intron_1/3_(13) | 65 | 0.011 |
| Laccaria_bicolor_H2B_4_Intron_2/3_(767) | Laccaria_bicolor_H2B_1_Intron_2/3_(767) | 3799 | 0 |
| Laccaria_bicolor_H2B_4_Intron_2/3_(767) | Laccaria_bicolor_H2B_5_Intron_2/3_(767) | 3612 | 0 |
| Laccaria_bicolor_H2B_4_Intron_3/3_(49) | Laccaria_bicolor_H2B_5_Intron_3/3_(49) | 245 | 7.30E-17 |
| Laccaria_bicolor_H2B_4_Intron_3/3_(49) | Laccaria_bicolor_H2B_1_Intron_3/3_(49) | 245 | 7.30E-17 |
| Laccaria_bicolor_H2B_5_Intron_1/3_(13) | Laccaria_bicolor_H2B_4_Intron_1/3_(13) | 65 | 0.011 |
| Laccaria_bicolor_H2B_5_Intron_1/3_(13) | Laccaria_bicolor_H2B_1_Intron_1/3_(13) | 65 | 0.011 |
| Laccaria_bicolor_H2B_5_Intron_2/3_(767) | Laccaria_bicolor_H2B_1_Intron_2/3_(767) | 3639 | 0 |
| Laccaria_bicolor_H2B_5_Intron_2/3_(767) | Laccaria_bicolor_H2B_4_Intron_2/3_(767) | 3612 | 0 |
| Laccaria_bicolor_H2B_5_Intron_3/3_(49) | Laccaria_bicolor_H2B_1_Intron_3/3_(49) | 245 | 7.30E-17 |
| Laccaria_bicolor_H2B_5_Intron_3/3_(49) | Laccaria_bicolor_H2B_4_Intron_3/3_(49) | 245 | 7.30E-17 |
| Aspergillus_fumigatus_H3_1_Intron_1/2_(129) | Neosartorya_fischeri_H3_1_Intron_1/2_(126) | 549 | 5.00E-35 |
| Aspergillus_fumigatus_H3_1_Intron_1/2_(129) | Aspergillus_oryzae_H3_1_Intron_1/2_(113) | 152 | 4.90E-06 |
| Aspergillus_fumigatus_H3_1_Intron_1/2_(129) | Aspergillus_niger_H3_1_Intron_1/2_(109) | 116 | 0.0021 |
| Aspergillus_fumigatus_H3_1_Intron_2/2_(53) | Neosartorya_fischeri_H3_1_Intron_2/2_(56) | 177 | 6.80E-10 |
| Aspergillus_fumigatus_H3_2_Intron_1/1_(74) | Neosartorya_fischeri_H3_2_Intron_1/1_(75) | 327 | 2.50E-18 |
| Aspergillus_nidulans_H3_1_Intron_1/2_(79) | Aspergillus_niger_H3_1_Intron_1/2_(109) | 109 | 0.005 |
| Aspergillus_niger_H3_1_Intron_1/2_(109) | Aspergillus_oryzae_H3_1_Intron_1/2_(113) | 181 | 1.10E-06 |
| Aspergillus_niger_H3_1_Intron_1/2_(109) | Aspergillus_fumigatus_H3_1_Intron_1/2_(129) | 116 | 0.0097 |
| Aspergillus_oryzae_H3_1_Intron_1/2_(113) | Aspergillus_niger_H3_1_Intron_1/2_(109) | 181 | 2.00E-07 |
| Aspergillus_oryzae_H3_1_Intron_1/2_(113) | Neosartorya_fischeri_H3_1_Intron_1/2_(126) | 176 | 4.30E-07 |
| Aspergillus_oryzae_H3_1_Intron_1/2_(113) | Aspergillus_fumigatus_H3_1_Intron_1/2_(129) | 152 | 1.70E-05 |
| Botryotinia_fuckeliana_H3_1_Intron_1/2_(164) | Sclerotinia_sclerotiorum_H3_1_Intron_1/2_(158) | 288 | 5.00E-17 |
| Botryotinia_fuckeliana_H3_1_Intron_1/2_(164) | Ustilago_maydis_H2A_1_Intron_1/1_(569) | 105 | 0.013 |
| Botryotinia_fuckeliana_H3_1_Intron_2/2_(51) | Sclerotinia_sclerotiorum_H3_1_Intron_2/2_(49) | 118 | 0.00021 |
| Botryotinia_fuckeliana_H3_2_Intron_1/2_(144) | Sclerotinia_sclerotiorum_H3_2_Intron_1/2_(147) | 128 | 0.0025 |
| Neosartorya_fischeri_H3_1_Intron_1/2_(126) | Aspergillus_fumigatus_H3_1_Intron_1/2_(129) | 549 | 7.80E-33 |
| Neosartorya_fischeri_H3_1_Intron_1/2_(126) | Aspergillus_oryzae_H3_1_Intron_1/2_(113) | 176 | 3.70E-07 |
| Neosartorya_fischeri_H3_1_Intron_1/2_(126) | Aspergillus_niger_H3_1_Intron_1/2_(109) | 109 | 0.015 |
| Neosartorya_fischeri_H3_1_Intron_2/2_(56) | Aspergillus_fumigatus_H3_1_Intron_2/2_(53) | 177 | 5.80E-09 |
| Neosartorya_fischeri_H3_2_Intron_1/1_(75) | Aspergillus_fumigatus_H3_2_Intron_1/1_(74) | 327 | 2.70E-19 |
| Podospora_anserine_H3_1_Intron_1/1_(81) | Podospora_anserine_H4_1_Intron_1/2_(297) | 134 | 0.0026 |
| Sclerotinia_sclerotiorum_H3_1_Intron_1/2_(158) | Botryotinia_fuckeliana_H3_1_Intron_1/2_(164) | 288 | 8.40E-15 |
| Sclerotinia_sclerotiorum_H3_1_Intron_2/2_(49) | Botryotinia_fuckeliana_H3_1_Intron_2/2_(51) | 118 | 0.0042 |
| Sclerotinia_sclerotiorum_H3_2_Intron_1/2_(147) | Botryotinia_fuckeliana_H3_2_Intron_1/2_(144) | 128 | 7.20E-05 |
| Laccaria_bicolor_H3_2_Intron_1/5_(137) | Laccaria_bicolor_H3_3_Intron_1/3_(175) | 276 | 2.50E-14 |
| Laccaria_bicolor_H3_2_Intron_4/5_(111) | Laccaria_bicolor_H3_3_Intron_3/3_(70) | 215 | 5.00E-09 |
| Laccaria_bicolor_H3_2_Intron_4/5_(111) | Laccaria_bicolor_H3_4_Intron_1/1_(68) | 160 | 1.40E-05 |
| Laccaria_bicolor_H3_3_Intron_1/3_(175) | Laccaria_bicolor_H3_2_Intron_1/5_(137) | 276 | 1.00E-16 |
| Laccaria_bicolor_H3_3_Intron_3/3_(70) | Laccaria_bicolor_H3_2_Intron_4/5_(111) | 215 | 1.10E-11 |
| Laccaria_bicolor_H3_3_Intron_3/3_(70) | Laccaria_bicolor_H3_4_Intron_1/1_(68) | 210 | 2.30E-11 |
| Laccaria_bicolor_H3_4_Intron_1/1_(68) | Laccaria_bicolor_H3_3_Intron_3/3_(70) | 210 | 7.10E-10 |
| Laccaria_bicolor_H3_4_Intron_1/1_(68) | Laccaria_bicolor_H3_2_Intron_4/5_(111) | 160 | 1.60E-06 |
| Aspergillus_fumigatus_H4_1_Intron_1/2_(71) | Neosartorya_fischeri_H4_2_Intron_1/2_(73) | 308 | 2.70E-16 |
| Aspergillus_fumigatus_H4_1_Intron_2/2_(55) | Neosartorya_fischeri_H4_2_Intron_2/2_(53) | 200 | 3.40E-09 |
| Aspergillus_fumigatus_H4_2_Intron_1/1_(24) | Neosartorya_fischeri_H4_1_Intron_1/1_(243) | 102 | 2.30E-04 |
| Botryotinia_fuckeliana_H4_1_Intron_1/2_(98) | Sclerotinia_sclerotiorum_H4_2_Intron_1/2_(108) | 279 | 2.30E-12 |
| Botryotinia_fuckeliana_H4_1_Intron_2/2_(55) | Sclerotinia_sclerotiorum_H4_2_Intron_2/2_(52) | 135 | 9.70E-06 |
| Botryotinia_fuckeliana_H4_2_Intron_1/4_(136) | Sclerotinia_sclerotiorum_H4_3_Intron_1/4_(139) | 185 | 3.50E-08 |
| Botryotinia_fuckeliana_H4_2_Intron_2/4_(51) | Sclerotinia_sclerotiorum_H4_3_Intron_2/4_(51) | 138 | 9.10E-07 |
| Botryotinia_fuckeliana_H4_2_Intron_3/4_(49) | Sclerotinia_sclerotiorum_H4_3_Intron_3/4_(49) | 110 | 0.00047 |
| Botryotinia_fuckeliana_H4_2_Intron_4/4_(58) | Sclerotinia_sclerotiorum_H4_3_Intron_4/4_(64) | 116 | 0.00018 |
| Botryotinia_fuckeliana_H4_3_Intron_1/2_(261) | Sclerotinia_sclerotiorum_H4_1_Intron_1/2_(237) | 275 | 2.70E-11 |
| Neosartorya_fischeri_H4_2_Intron_1/2_(73) | Aspergillus_fumigatus_H4_1_Intron_1/2_(71) | 308 | 1.90E-16 |
| Neosartorya_fischeri_H4_2_Intron_2/2_(53) | Aspergillus_fumigatus_H4_1_Intron_2/2_(55) | 200 | 9.00E-10 |
| Podospora_anserine_H4_1_Intron_1/2_(297) | Podospora_anserine_H3_1_Intron_1/1_(81) | 134 | 0.0066 |
| Sclerotinia_sclerotiorum_H4_1_Intron_1/2_(237) | Botryotinia_fuckeliana_H4_3_Intron_1/2_(261) | 275 | 9.60E-12 |
| Sclerotinia_sclerotiorum_H4_2_Intron_1/2_(108) | Botryotinia_fuckeliana_H4_1_Intron_1/2_(98) | 279 | 2.00E-12 |
| Sclerotinia_sclerotiorum_H4_2_Intron_2/2_(52) | Botryotinia_fuckeliana_H4_1_Intron_2/2_(55) | 135 | 6.70E-05 |
| Sclerotinia_sclerotiorum_H4_3_Intron_1/4_(139) | Botryotinia_fuckeliana_H4_2_Intron_1/4_(136) | 185 | 1.30E-07 |
| Sclerotinia_sclerotiorum_H4_3_Intron_2/4_(51) | Botryotinia_fuckeliana_H4_2_Intron_2/4_(51) | 138 | 2.90E-07 |
| Sclerotinia_sclerotiorum_H4_3_Intron_3/4_(49) | Botryotinia_fuckeliana_H4_2_Intron_3/4_(49) | 110 | 0.00034 |
| Sclerotinia_sclerotiorum_H4_3_Intron_4/4_(64) | Botryotinia_fuckeliana_H4_2_Intron_4/4_(58) | 116 | 0.00021 |
| Laccaria_bicolor_H4_2_Intron_1/1_(55) | Laccaria_bicolor_H4_5_Intron_3/3_(57) | 183 | 2.90E-09 |
| Laccaria_bicolor_H4_2_Intron_1/1_(55) | Laccaria_bicolor_H4_4_Intron_3/3_(55) | 176 | 9.80E-09 |
| Laccaria_bicolor_H4_3_Intron_1/2_(45) | Laccaria_bicolor_H4_8_Intron_1/2_(45) | 225 | 1.60E-10 |
| Laccaria_bicolor_H4_3_Intron_2/2_(427) | Laccaria_bicolor_H4_8_Intron_2/2_(443) | 2120 | 7.30E-161 |
| Laccaria_bicolor_H4_4_Intron_2/3_(51) | Laccaria_bicolor_H4_9_Intron_1/1_(51) | 165 | 3.90E-09 |
| Laccaria_bicolor_H4_4_Intron_3/3_(55) | Laccaria_bicolor_H4_5_Intron_3/3_(57) | 210 | 2.20E-10 |
| Laccaria_bicolor_H4_4_Intron_3/3_(55) | Laccaria_bicolor_H4_2_Intron_1/1_(55) | 176 | 5.70E-08 |
| Laccaria_bicolor_H4_5_Intron_3/3_(57) | Laccaria_bicolor_H4_4_Intron_3/3_(55) | 210 | 6.00E-10 |
| Laccaria_bicolor_H4_5_Intron_3/3_(57) | Laccaria_bicolor_H4_2_Intron_1/1_(55) | 183 | 4.30E-08 |
| Laccaria_bicolor_H4_6_Intron_1/2_(55) | Laccaria_bicolor_H4_7_Intron_1/3_(55) | 266 | 2.60E-15 |
| Laccaria_bicolor_H4_6_Intron_1/2_(55) | Laccaria_bicolor_H4_11_Intron_1/3_(55) | 257 | 1.20E-14 |
| Laccaria_bicolor_H4_6_Intron_1/2_(55) | Laccaria_bicolor_H4_10_Intron_1/3_(54) | 119 | 0.00028 |
| Laccaria_bicolor_H4_6_Intron_2/2_(61) | Laccaria_bicolor_H4_7_Intron_2/3_(61) | 296 | 2.90E-21 |
| Laccaria_bicolor_H4_6_Intron_2/2_(61) | Laccaria_bicolor_H4_11_Intron_2/3_(60) | 248 | 5.70E-17 |
| Laccaria_bicolor_H4_7_Intron_1/3_(55) | Laccaria_bicolor_H4_6_Intron_1/2_(55) | 266 | 1.10E-15 |
| Laccaria_bicolor_H4_7_Intron_1/3_(55) | Laccaria_bicolor_H4_11_Intron_1/3_(55) | 248 | 2.60E-14 |
| Laccaria_bicolor_H4_7_Intron_1/3_(55) | Laccaria_bicolor_H4_10_Intron_1/3_(54) | 110 | 0.001 |
| Laccaria_bicolor_H4_7_Intron_2/3_(61) | Laccaria_bicolor_H4_6_Intron_2/2_(61) | 296 | 5.80E-21 |
| Laccaria_bicolor_H4_7_Intron_2/3_(61) | Laccaria_bicolor_H4_11_Intron_2/3_(60) | 239 | 6.20E-16 |
| Laccaria_bicolor_H4_7_Intron_3/3_(53) | Laccaria_bicolor_H4_11_Intron_3/3_(53) | 229 | 4.80E-13 |
| Laccaria_bicolor_H4_8_Intron_1/2_(45) | Laccaria_bicolor_H4_3_Intron_1/2_(45) | 225 | 1.60E-10 |
| Laccaria_bicolor_H4_8_Intron_2/2_(443) | Laccaria_bicolor_H4_3_Intron_2/2_(427) | 2120 | 1.20E-160 |
| Laccaria_bicolor_H4_9_Intron_1/1_(51) | Laccaria_bicolor_H4_4_Intron_2/3_(51) | 165 | 2.30E-07 |
| Laccaria_bicolor_H4_10_Intron_1/3_(54) | Laccaria_bicolor_H4_6_Intron_1/2_(55) | 119 | 4.70E-05 |
| Laccaria_bicolor_H4_10_Intron_1/3_(54) | Laccaria_bicolor_H4_11_Intron_1/3_(55) | 110 | 0.00027 |
| Laccaria_bicolor_H4_10_Intron_1/3_(54) | Laccaria_bicolor_H4_7_Intron_1/3_(55) | 110 | 0.00027 |
| Laccaria_bicolor_H4_11_Intron_1/3_(55) | Laccaria_bicolor_H4_6_Intron_1/2_(55) | 257 | 9.60E-16 |
| Laccaria_bicolor_H4_11_Intron_1/3_(55) | Laccaria_bicolor_H4_7_Intron_1/3_(55) | 248 | 5.00E-15 |
| Laccaria_bicolor_H4_11_Intron_1/3_(55) | Laccaria_bicolor_H4_10_Intron_1/3_(54) | 110 | 0.00051 |
| Laccaria_bicolor_H4_11_Intron_2/3_(60) | Laccaria_bicolor_H4_6_Intron_2/2_(61) | 248 | 1.40E-15 |
| Laccaria_bicolor_H4_11_Intron_2/3_(60) | Laccaria_bicolor_H4_7_Intron_2/3_(61) | 239 | 8.10E-15 |
| Laccaria_bicolor_H4_11_Intron_3/3_(53) | Laccaria_bicolor_H4_7_Intron_3/3_(53) | 229 | 8.30E-15 |
